# Efficacy and safety of tiotropium in COPD patients in primary care – the SPiRiva Usual CarE (SPRUCE) study

**DOI:** 10.1186/1465-9921-8-45

**Published:** 2007-07-02

**Authors:** Daryl Freeman, Angela Lee, David Price

**Affiliations:** 1 Department of General Practice and Primary Care, University of Aberdeen, Aberdeen, UK; 2 Independent Statistician, UK

## Abstract

**Background:**

Clinical trials of tiotropium have principally recruited patients from secondary care with more severe chronic obstructive pulmonary disease (COPD), and typically had included limitation of concomitant medication. In primary care, which is the most common setting for COPD management, many patients may have milder disease, and also may take a broad range of concomitant medication.

**Methods:**

This randomised, placebo-controlled, parallel-group, 12-week, 44-centre study investigated the efficacy (trough forced expiratory volume in 1 second [FEV_1_] response) and safety of additional treatment with once-daily tiotropium 18 μg via the HandiHaler^® ^in a primary care COPD population (tiotropium: N = 191, FEV_1 _= 1.25 L [47.91% predicted]; placebo: N = 183, FEV_1 _= 1.32 L [49.86% predicted]). Secondary endpoints included: trough forced vital capacity (FVC) response, weekly use of rescue short-acting β-agonist, and exacerbation of COPD (complex of respiratory symptoms/events of >3 days in duration requiring a change in treatment). Treatment effects were determined using non-parametric analysis.

**Results:**

At Week 12, median improvement in trough FEV_1 _response with tiotropium versus placebo was 0.06 L (p = 0.0102). The improvement was consistent across baseline treatment and COPD severity. Median improvement in FVC at 2, 6 and 12 weeks was 0.12 L (p < 0.001). The percentage of patients with ≥1 exacerbation was reduced (tiotropium 9.5%; placebo 17.9%; p = 0.0147), independent of disease severity. Rescue medication usage was significantly reduced in the tiotropium group compared with placebo. Adverse event profile was consistent with previous studies.

**Conclusion:**

Tiotropium provides additional benefits to usual primary care management in a representative COPD population.

**Trial registration:**

The identifier is: NCT00274079.

## Background

Chronic obstructive pulmonary disease (COPD) is increasingly recognised as a significant burden to patients and the health economy. As a result, national and international guidelines on management have been introduced for the diagnosis and management of this disease [1-4]. The implementation of diagnosis and treatment guidelines in primary care has been a key strategy for national agencies [2]. COPD is still under-diagnosed and the role of primary care is critical to early and correct diagnosis, since a large proportion of smokers with early symptoms of COPD are first seen by their primary care physician [5-7].

Bronchodilators are the first-line approach to treatment in patients with COPD of all severities [1-4]. Bronchodilation in COPD is believed to be achieved largely through inhibition of the smooth muscle tone maintained in the airways by the parasympathetic nervous system [8].

Tiotropium is a once-daily, long-acting anticholinergic that acts through prolonged M_3_-receptor blockade [9,10]. It has been widely investigated prior to registration in secondary care settings in patients with COPD [11]. Several large studies have demonstrated that tiotropium significantly improves lung function when compared with placebo, the short-acting anticholinergic ipratropium bromide, or a long-acting β-agonist (LABA) [11,12,14]. In addition, tiotropium reduces dyspnoea, lowers rescue medication use, improves health-related quality of life, reduces the incidence and number of exacerbations, and delays the time to both first exacerbation and first hospitalisation compared with either placebo or ipratropium [11,12].

These previous studies with tiotropium [11-14] recruited patients predominantly from secondary care clinics with moderate-to-severe disease according to the National Institute for Health and Clinical Excellence (NICE) guidelines [2] and severe-to-very severe according to the Global Initiative for Chronic Obstructive Lung Disease (GOLD) [3]. Furthermore, LABAs were not allowed during the treatment period. Hence, while these are important studies for secondary care, neither the patients nor the treatment practices are representative of experiences in primary care.

The current study was designed to assess the effects of introducing tiotropium to usual treatment in conditions representative of normal clinical practice. It included patients defined by their general practitioner as having COPD, with a broad range of disease severity, and with a broad range of other treatments, including LABAs and inhaled corticosteroids (ICS).

## Methods

### Patients

Patients were required to have a COPD diagnosis according to British Thoracic Society criteria [1] and recent stable disease (no exacerbation or respiratory infection within 4 weeks), with airway obstruction forced expiratory volume in 1 second (FEV_1_) between 30% and 65% of predicted normal value and FEV_1_/forced vital capacity (FVC) ≤70% pre-bronchodilators. Patients with a history of allergy or asthma were excluded. Predicted normal values were calculated according to European Coal and Steel Community (ECSC) [15]. Patients had to be at least 40 years old, have at least a 10 pack-year smoking history and had to be receiving short-acting β_2_-agonists (SABA) as rescue medication (salbutamol or terbutaline metered dose inhaler [MDI] or dry powder inhaler [DPI]) with no anticholinergic drug prescribed in the preceding year. Patients had to be able to undergo spirometry and be able to use the HandiHaler^® ^device.

Patients were excluded if they had any other significant medical condition that might interfere with the study or preclude their use of study medication, such as known hypersensitivity to anticholinergic drugs, known symptomatic prostatic hypertrophy, narrow angle glaucoma, severe cardiovascular disease, or recent myocardial infarction (≤1 year). Patients who were on long-term oxygen therapy (LTOT) were also excluded.

### Study design

Forty-four primary care centres throughout England, Scotland and Wales participated in this 12-week, multi-centre, randomised, double-blind, placebo-controlled, parallel-group study (Study #205.276). The study was led by an independent steering committee comprising the study authors and was approved by national and regional ethical committees. Written informed consent was obtained from all patients before the study procedure was undertaken.

At the screening visit, demographic data, smoking history and COPD characteristics were collected. A full medical history and physical examination, including vital signs (blood pressure and pulse rate) and 6- or 12-lead electrocardiogram (ECG), were conducted. Pulmonary function tests (PFTs) (FEV_1 _and FVC) were conducted in the morning between 8:00 am and 11:00 am at all visits.

Following screening, patients entered a 2-week run-in period, during which their COPD had to remain stable (i.e. no exacerbations for 6 weeks). At the baseline visit (14 ± 2 days after the screening visit), patients were randomised to 12-week treatment with either tiotropium 18 μg or placebo (1:1 block randomisation), in addition to their usual treatment. Medication and placebo were delivered by identical-appearing lactose-based inhalers (HandiHaler^®^, Boehringer Ingelheim, Ingelheim am Rhein, Germany). FEV_1 _and FVC measurements were performed on Week 2 (Day 15), Week 6 (Day 43) and Week 12 (Day 85), at the same time of day (± 30 minutes) as assessments during the baseline visit.

### Measurements

The primary efficacy endpoint was trough FEV_1 _response at the end of the 12-week treatment period. Trough FEV_1 _response was defined as the change from baseline at the end of the 24-hour dosing interval (i.e. 10 minutes prior to drug administration). Baseline FEV_1 _was the pre-treatment FEV_1 _measured at Visit 2, 10 minutes prior to administration of the first dose of the study medication.

The Micro Medical Lab 2000 spirometer was used by all centres. The spirometers and their use, including calibration, had to meet the American Thoracic Society's criteria [16]. Spirometry training was given prior to initiating the study and a repeat training session was given during the initiation visit. Spot checks on the calibration of spirometers were conducted during the monitoring visits and randomly selected spirographs were inspected by four independent reviewers from within the steering committee after the study, to confirm that acceptable quality curves had been produced. Acceptability/non-acceptability of the lung function curves was assigned by consensus.

Patients were asked not to take their morning respiratory medications (according to a pre-established washout period) prior to the morning PFT. The highest value of FEV_1 _and FVC from three technically acceptable manoeuvres were recorded [12].

Secondary spirometry endpoints included trough FEV_1 _response after 2 and 6 weeks, and trough FVC response after 2, 6 and 12 weeks.

SABA use was recorded daily by the patient in a diary card, and mean uses per day were calculated on a weekly basis.

At all visits, all adverse events, serious and non-serious, and regardless of causality, were collected. Adverse event records were used to identify COPD exacerbations. An exacerbation of COPD was defined as a complex of respiratory events/symptoms with duration of 3 or more days (from patient's diary card) requiring a change in treatment (including patient-initiated increases). A complex of respiratory events/symptoms meant ≥2 of the following (increase of symptom or new onset): shortness of breath, sputum production (volume), cough, wheezing and chest tightness. The change in (or requirement of) treatment included prescription of antibiotics and/or systemic steroids and/or a significant change (including increase) of the prescribed respiratory medication (bronchodilators including theophylline).

Dyspnoea was measured by the Oxygen Cost Diagram (OCD) as an exploratory outcome. The OCD is a visual analogue scale with 13 activities listed along a 100 mm line [17]. Patients were asked at each visit to indicate on the line the level of activity at which they started to experience dyspnoea. This measurement was included to examine standard deviation for use in future clinical trials in various categories of COPD patients.

The inhalation powder capsules (used and unused) were counted to assess treatment compliance.

### Statistical analysis

The primary objective was to determine the effect on lung function when either tiotropium 18 μg inhalation capsules or placebo was added to the usual therapy of COPD patients managed in primary care who had not received inhaled anticholinergics during the previous 12 months.

In previous studies of COPD patients who were not on LABAs, the standard deviation (SD) for trough FEV_1 _was 215 ml and a tiotropium effect size of 130 ml [11]. It was assumed that 20% of patients with COPD who are managed in primary care would be using LABAs as part of their usual care, and that the effect of tiotropium on mean trough FEV_1 _in the study population would be lower than the 130 ml seen in previous studies. Placebo was expected to have no effect on mean trough FEV_1_. Assuming an SD of 235 ml, a total of 348 patients (174 per group) was determined to be adequate to detect a difference of 100 ml in trough FEV_1 _response between treatments with at least 96% power at the 5% level of significance (two-sided) using a two-group *t*-test.

Analysis of all endpoints was planned with treatment differences evaluated using analysis of covariance. Due to significant skewing with heavy tails seen in the distribution of the primary endpoint (trough FEV_1 _response) in both treatment groups, a non-parametric approach was considered appropriate by the steering committee for the primary endpoint and, for consistency, was applied to all the efficacy endpoints. The Mann-Whitney test and Hodges-Lehman shift parameter for effect size estimate and 95% confidence interval (CI) were therefore used to compare the treatments.

The number and percent of patients with ≥1 COPD exacerbation (MedDRA preferred term) was compared using the Chi-square test.

To include as many patients as possible, efficacy analyses were performed using the Full Analysis Set (FAS), following the intent-to-treat principle – i.e. randomised patients with both baseline data and data following multiple doses of randomised treatment – for PFT and diary cards (DIARY). Missing data due to worsening of COPD were imputed using the least favourable data model. Data missing for other reasons were imputed with the last observed data model.

## Results

### Patients

**Figure 1:**
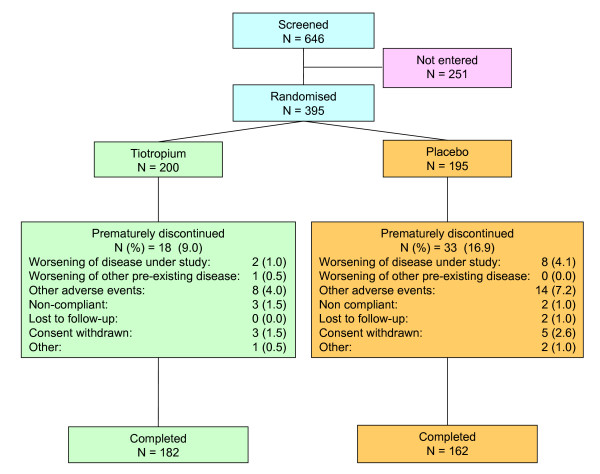
Study flow.

Of the 646 patients screened for entry into the study, 251 (38.9%) were not eligible. A high proportion of the screened patients (165; 25.5%) failed the lung function entry criteria (30% <FEV_1 _≤65% and FEV_1_/FVC ratio ≤70%). Of these 25.5% failing entry, 5.6% had ≤30% of FEV_1 _predicted, 57.1% had >65% of FEV_1 _predicted, and 45.1% had an FEV_1_/FVC ratio >70%. Of the remaining 395 patients, 200 were randomly assigned to tiotropium and 195 to placebo. Forty-four centres across the UK participated in the study, which lasted 12 months from October 2002 to October 2003 (Fig. 1).

Demographics and baseline characteristics of all randomised patients in the FAS for PFTs are shown in Table 1. Compliance with study medication based on capsule counts was high and similar in the two treatment groups (99%).

**Table 1:** Demographics and baseline values* of FAS-PFT randomised patients

	**Tiotropium**	**Placebo**	**Total**
**Total randomised (N)**	**191**	**183**	**374**
Male, n (%)	96 (50.3)	107 (58.5)	203 (54.3)
Age (years)	64.7 (9.0)	65.1 (9.3)	64.9 (9.1)
Smoking history (pack-years)	36.9 (16.9)	37.9 (17.7)	37.4 (17.3)
*Screening (Visit 1)*			
FEV_1 _(L) pre-bronchodilator	1.25 (0.42)	1.32 (0.44)	1.28 (0.43)
FEV_1 _% predicted normal	47.91 (10.49)	49.86 (10.71)	48.86 (10.63)
FEV_1_/FVC %	55.24 (9.69)	55.79 (10.01)	55.51 (9.84)
FVC (L)	2.30 (0.75)	2.41 (0.84)	2.36 (0.80)
*NICE classification of COPD severity of obstruction, n (%)[2]*			
Mild (FEV_1 _≥50% predicted)	86 (45.0)	92 (50.3)	178 (47.6)
Moderate (30% ≤FEV_1 _<50% predicted)	97 (50.8)	88 (48.1)	185 (49.5)
Severe (FEV_1 _<30% predicted)	8 (4.2)	3 (1.6)	11 (2.9)
*Pulmonary medication during baseline^†^*			
LABA, n (%)	55 (28.8)	53 (29.0)	108 (28.9)
LABA, no ICS, n (%)	6 (3.1)	7 (3.8)	13 (3.5)
ICS, n (%)	124 (64.9)	113 (61.8)	237 (63.4)
ICS, no LABA, n (%)	75 (39.3)	67 (36.6)	142 (38.0)
LABA plus ICS, n (%)	49 (25.7)	46 (25.1)	95 (25.4)
No LABA, no ICS, n (%)	61 (31.9)	63 (34.4)	124 (33.2)
SABA use, occasions/week^‡^	3.83 (2.47)	3.52 (2.51)	3.68 (2.49)

* Mean (SD) unless otherwise stated

^† ^Baseline pulmonary medication were those started before informed consent and included those ended on or after the consent date

^‡ ^Mean (SD) number of occasions of SABA use during the last week of the baseline period

Definitions of abbreviations: FAS-PFT = Full Analysis Set-pulmonary function tests; FEV_1 _= forced expiratory volume in 1 second; FVC = forced vital capacity; NICE = National Institute of Clinical Excellence; COPD = chronic obstructive pulmonary disease; LABA = long-acting β-agonist; SABA = short-acting β-agonist; ICS = inhaled corticosteroid.

### Quality of spirometry

A total of 272 curves (52 randomised patients), representing 20% of the curves both from low-recruiting centres (<6 patients; n = 8) and high-recruiting centres (>20 patients; n = 4) were selected. Of the 272 curves, 223 curves (82%) were considered acceptable, 44 (16%) were considered unacceptable, and 5 (2%) of the curves were too faded to read during the audit. Exclusion of unacceptable/unreadable curves did not influence the results of the analysis of this study.

### Primary endpoint

On Day 85, the median trough FEV_1 _response was 0.09 L in the tiotropium group and 0.03 L in the placebo group (Table 2). The distribution of results showed significant skew, with 27 patients in the tiotropium group and 23 in the placebo group having an increase in FEV_1 _≥0.4 L.

**Table 2:** Median (interquartile range) for trough FEV_1 _and FVC responses (L) on test days (FAS-PFT)

**Test day**	**Tiotropium (N = 191)**	**Placebo (N = 183)**	**p value**	Effect size* (95% CI)
*Trough FEV_1 _response^†^*				
15	0.07 (-0.03, 0.19)	0.00 (-0.08, 0.12)	0.0036	0.06 (0.02, 0.10)
43	0.08 (-0.04, 0.21)	0.04 (-0.08, 0.16)	NS	0.03 (-0.02, 0.07)
85	0.09 (-0.03, 0.28)	0.03 (-0.07, 0.14)	0.0102	0.06 (0.01, 0.10)
*Trough FVC response^†^*				
15	0.11 (-0.06, 0.26)	-0.03 (-0.18, 0.14)	<0.0001	0.12 (0.07, 0.17)
43	0.12 (-0.06, 0.29)	-0.02 (-0.19, 0.19)	0.0001	0.12 (0.06, 0.18)
85	0.09 (-0.08, 0.33)	0.01 (-0.17, 0.17)	0.0002	0.12 (0.05, 0.18)

* Hodges-Lehman shift parameter estimate with 95% CI

^† ^Response is change from baseline

Definitions of abbreviations: FEV_1 _= forced expiratory volume in 1 second; FVC = forced vital capacity; FAS-PFT = Full Analysis Set-pulmonary function tests; CI = confidence interval.

Regarding trough FEV_1 _response following 12 weeks on randomised treatment (Day 85), an improvement of 0.06 L (95% CI: 0.01 L, 0.10 L) was seen with tiotropium compared with placebo. The difference was statistically significant (p = 0.0102) (Table 2).

### Secondary endpoints

Trough FEV_1 _response also showed an improvement with tiotropium compared with placebo on Days 15 and 43, but did not reach significance on Day 43 (Table 2). Trough FVC responses were significantly better with tiotropium compared with placebo on all test days (p < 0.001) (Table 2).

Subgroup analysis according to use of LABA, ICS or combination (either fixed-dose or free-dose combinations) generally showed numerical trends for improvements with tiotropium compared with placebo in both FEV_1 _and FVC trough responses on Day 85, though effect sizes were small (Table 3).

**Table 3:** Median (interquartile range) for trough FEV_1 _and FVC responses on Day 85 according to LABA, ICS or combination use during the treatment period (FAS-PFT)*

	**Trough FEV_1 _response (L)^†^**			**Trough FVC response (L)^†^**		
	
	**Tiotropium**	**Placebo**	**Effect size^‡^**	**Tiotropium**	**Placebo**	**Effect size^‡^**
**LABA **(tiotropium n = 56; placebo n = 55)	0.05 (-0.07, 0.17)	-0.01 (-0.09, 0.07)	0.07 (0.00, 0.14)	0.05 (-0.12, 0.32)	-0.05 (-0.21, 0.08)	0.12 (0.02, 0.23)
**ICS **(tiotropium n = 126, placebo n = 113)	0.12 (-0.05, 0.26)	0.00 (-0.09, 0.11)	0.09 (0.04, 0.15)	0.09 (-0.08, 0.35)	-0.01 (-0.17, 0.14)	0.14 (0.06, 0.22)
**ICS; no LABA **(tiotropium n = 76, placebo n = 65)	0.14 (-0.02, 0.33)	0.02 (-0.08, 0.14)	0.09 (0.01, 0.18)	0.11 (-0.08, 0.43)	0.02 (-0.18, 0.17)	0.14 (0.03, 0.26)
**LABA plus ICS **(tiotropium n = 50, placebo n = 48)	0.06 (-0.07, 0.19)	-0.02 (-0.09, 0.07)	0.09 (0.01, 0.17)	0.05 (-0.09, 0.34)	-0.04 (-0.18, 0.08)	0.13 (0.02, 0.26)
**No LABA; no ICS **(tiotropium n = 59, placebo n = 63)	0.09 (-0.02, 0.31)	0.09 (-0.01, 0.27)	0.01 (-0.07, 0.09)	0.13 (-0.06, 0.30)	0.06 (-0.14, 0.21)	0.08 (-0.02, 0.19)

* Numbers of patients for LABA, no ICS were too small for any meaningful comparison (tiotropium group, n = 6; placebo group, n = 7)

^† ^Response is change from baseline

^‡ ^Hodges-Lehman shift parameter estimate with 95% CI

Definitions of abbreviations: FEV_1 _= forced expiratory volume in 1 second; FVC = forced vital capacity; LABA = long-acting β-agonist; ICS = inhaled corticosteroid; FAS-PFT = Full Analysis Set-pulmonary function tests.

Analysis of data in the subgroup of patients with mild COPD (FEV_1 _≥50% predicted, according to NICE classification [2]), suggested a small but significant improvement in the trough FVC response with tiotropium compared with placebo on Day 85 (Table 4). A similar trend was shown for the trough FEV_1 _response, though this did not achieve statistical significance in this subgroup analysis (Table 4). Tiotropium significantly improved both trough FEV_1 _and trough FVC responses compared with placebo in patients with moderate or worse COPD (FEV1 <50% predicted).

**Table 4:** Median (interquartile range) for trough FEV_1 _and FVC responses (L) on Day 85 according to patients with either FEV_1 _≥50% predicted or FEV_1 _<50% predicted (FAS-PFT)

	**Trough FEV_1 _response (L)^†^**			**Trough FVC response (L)^†^**		
	
	**Tiotropium**	**Placebo**	**Effect size^†^**	**Tiotropium**	**Placebo**	**Effect size^†^**
**FEV_1 _≥50% predicted ** (tiotropium n = 86; placebo n = 92)	0.06 (-0.08, 0.23)	0.03 (-0.08, 0.12)	0.04 (-0.03, 0.10)	0.07 (-0.12, 0.22)	-0.01 (-0.18, 0.12)	0.08^‡ ^ (0.00, 0.16)
**FEV_1 _<50% predicted** (tiotropium n = 105, placebo n = 91)	0.13 (-0.02, 0.30)	0.02 (-0.07, 0.17)	0.07^‡ ^ (0.01, 0.14)	0.17 (-0.05, 0.42)	0.02 (-0.14, 0.18)	0.14^‡ ^ (0.05, 0.24)

* Response is change from baseline

^† ^Hodges-Lehman shift parameter estimate with 95% CI

^‡ ^p < 0.05

Definitions of abbreviations: FEV_1 _= forced expiratory volume in 1 second; FVC = forced vital capacity; FAS-PFT = Full Analysis Set-pulmonary function tests; CI = confidence interval.

**Figure 2:**
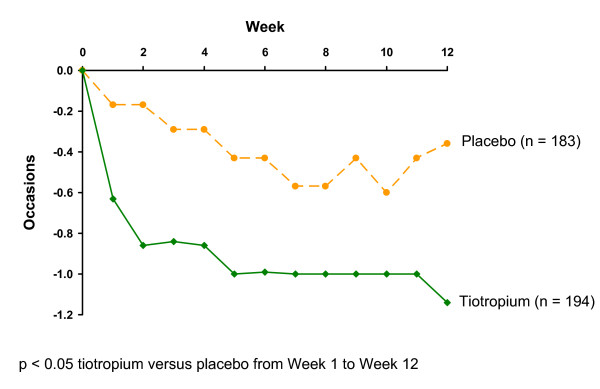
Median changes from baseline in weekly mean number of occasions per day of rescue SABA use over 12 weeks (FAS-DIARY).

The use of rescue medication was significantly lower for tiotropium compared with placebo throughout the 12-week treatment period (p < 0.05) (Fig. 2). Significantly fewer tiotropium patients (n = 19; 9.5%) experienced one or more COPD exacerbations than placebo (n = 35; 17.9%) (p = 0.0147) (Table 5). Subanalysis of COPD exacerbations by severity of airflow obstruction according to NICE criteria at baseline also showed a numerical trend towards lower incidence in the tiotropium group compared with placebo across severity categories (Table 5). The changes in treatment that partly defined an exacerbation were mostly changes in antibiotic and/or oral steroid treatment (Table 5).

**Table 5:** COPD exacerbations by treatment and according to baseline disease severity during the trial in randomised patients

	**Tiotropium**	**Placebo**
Number of patients treated	200	195
≥1 COPD exacerbation, n (%)	19 (9.5)	35 (17.9)*
1 exacerbation, n (%)	15 (7.5)	28 (14.4)
2 exacerbations, n (%)	4 (2.0)	5 (2.6)
3 exacerbations, n (%)	0 (0.0)	2 (1.0)
Baseline disease severity, proportion (%)		
Mild (FEV_1 _≥50% predicted)	6/88 (6.8)	16/95 (16.8)*
Moderate (30% ≤FEV_1 _<50% predicted)	11/104 (10.6)	18/96 (18.8)
Severe (FEV_1 _<30% predicted)	2/8 (25.0)	1/4 (25.0)

Moderate/severe (FEV_1 _<50% predicted)	13/112 (11.6)	19/100 (19.0)

Treatment change, n (%)^†^		
Antibiotics	12 (6.0)	13 (6.7)
+ bronchodilator		4 (2.0)
+ bronchodilator + oral steroid	1 (0.5)	1 (0.5)
+ oral steroid	2 (1.0)	12 (6.2)
Oral steroid		3 (1.5)
Bronchodilator	1 (0.5)	1 (0.5)
+ inhaled steroid	3 (1.5)	
Inhaled steroid		1 (0.5)

* p < 0.05

^† ^Treatment change included prescription of antibiotics and/or systemic steroids and/or a significant change (including increase) of the prescribed respiratory medication (bronchodilators including theophylline)

Definitions of abbreviations: COPD = chronic obstructive pulmonary disease; FEV_1 _= forced expiratory volume in 1 second

### Exploratory outcome

There was no significant increase in OCD score in the tiotropium compared with the placebo group. The median change (interquartile range) from baseline at Day 85 was 3.5 mm (-2.0, 20.5) with tiotropium (maximum change of 57.0 mm) and 3.5 mm (-1.0, 20.0) with placebo (maximum change of 61.0 mm).

### Safety

Adverse events were reported in 51.0% of patients treated with tiotropium, and 61.5% of patients receiving placebo. Higher incidences of lower respiratory system disorders were observed in the placebo group (30.8%) compared with tiotropium (23.0%).

Dry mouth was the most frequently reported drug-related adverse event that was higher in the tiotropium group (5.5%) compared with placebo (1.5%).

## Discussion

This study, which is the first study with tiotropium conducted in primary care, was conducted to assess the efficacy and safety of tiotropium when introduced to usual primary care management across a broad COPD population. A significant improvement with tiotropium was seen compared with placebo in bronchodilator response, assessed by trough FEV_1 _and FVC, with good tolerability. Although the magnitude of the improvement was small, significance in the primary outcome and many secondary outcomes was achieved.

The lung function results, along with the reduction in use of rescue medication and COPD exacerbations, are in agreement with studies of tiotropium with patient populations seemingly recruited from secondary care centres [11-14]. The majority of patients with COPD receive treatment mainly from primary care; hence, it is probable that many patients recruited in studies from secondary centres would overlap with the patients recruited in our study, which was designed to be representative of normal clinical practice. In this respect, patients with milder disease by GOLD staging were reported in these and other secondary care studies with tiotropium [18,19], and this subgroup of patients respond to tiotropium with respect to improved lung function, health status and need for rescue medication.

On the whole, patients recruited to our study, in addition to frequently receiving concomitant medication with LABA and/or ICS, differed from those in earlier studies because they had somewhat higher mean pre-bronchodilator FEV_1 _% predicted (49%, compared with 38% to 42% in earlier studies) and FEV_1_/FVC (56%, compared with 42% to 46% in earlier studies) [11]. These results therefore both confirm and extend the findings of previous studies with tiotropium.

Considerable skewing of data was seen in the distribution of the primary endpoint, trough FEV_1 _response, in both treatment groups, and this has resulted in non-parametric analyses being used for the study. The most likely explanation for this skewed response lies in the many patients who showed an unusually large response to treatment, including an unexpected number of patients with sizable improvements in both treatment groups. This occurred despite entry criteria selecting patients on smoking history, obstruction and no prior history of allergy or asthma, and illustrates the difficulty of excluding all patients who have a reversible component to their disease, especially in those with milder disease. Although this resulted in the skewing of data, the authors would argue that this study design represents what happens in normal clinical practice – and indeed is at present endorsed by NICE, who suggest reversibility testing is not a requirement for a routine diagnosis of COPD [2]. The results of this study suggest this advice requires further study, especially in those with milder airflow restriction.

Tiotropium showed a significant improvement in trough FEV_1 _on Days 15 and 85, and a larger improvement in trough FVC at all data collection points. Subgroup analysis of patients with mild COPD [2] suggested a similar trend on Day 85, with a more marked improvement with tiotropium in trough FVC response compared with trough FEV_1 _response. The clinical significance of this is likely to be related to reduction in air trapping, as has already been demonstrated in specific studies of shorter duration [20,21]. O'Donnell *et al*., in a 42-day study, demonstrated that tiotropium produced sustained reductions of lung hyperinflation at rest and during exercise [20]. In a 4-week study in COPD patients with increased static lung volumes, Celli *et al*. showed that, compared with placebo, tiotropium improved inspiratory capacity (IC) and reduced total gas volume (TGV) [21]. The authors of both studies concluded that increases in IC permitted greater expansion of tidal volume and contributed to improvements in both exertional dyspnoea and exercise endurance.

The proportion of patients on LABAs with ICS was higher than expected and this could have had some influence on the outcomes of this study. The additional bronchodilator effect of tiotropium when given to patients on LABAs has been reported [22-24]. However, this could not be investigated in subgroup analyses of this study as most patients on LABAs were also treated with ICS. Nevertheless, the bronchodilator response to tiotropium was maintained with concomitant use of LABAs and/or ICS in subgroup analyses, consistent with the Canadian Optimal Therapy of COPD Trial [25]. Hence, this provides further support to the notion that tiotropium provides additional and sustained efficacy to usual care. Unexpectedly, patients in the placebo group who were not treated with either LABAs or ICS had improved FEV_1 _and FVC responses at the end of the trial compared with baseline. There was nothing remarkable about this subgroup compared with the other subgroups that would explain this result with our data from a small sample. However, if this result is truly representative, it may be that patients untreated with LABAs or ICS are milder than those receiving these interventions.

A significant reduction in COPD exacerbations was observed, despite the relatively short-term duration of the study (12 weeks), and the small sample size. This reduction is consistent with longer studies [11-14], the clinical relevance and cost effectiveness of which has already been demonstrated [26,27]. Although the definition of COPD exacerbations is still under discussion, there is a general consensus that acute exacerbations should be defined based on symptoms (worsening or new) and need for medical intervention [28,29]. The definition of COPD exacerbation used in this study meets these criteria [11-14].

The reduction in the use of rescue medication observed in this study is a consistent finding in all tiotropium studies [11-14] and supports the clinical relevance of the sustained efficacy of tiotropium and reduction of COPD exacerbations. The actual magnitude of the reduction in rescue medication in this study is smaller in absolute terms than in previous studies, but this could have been anticipated considering the generally more mild patients with lower baseline usage and the permitted use of LABAs throughout the study. The fact that there was a reduction in rescue medication with no meaningful change on the OCD suggests that the patients were able to accurately self-medicate to maintain their symptoms at an acceptable level; although the limited sensitivity of the OCD could be a factor in this result [30]. Future trials need to be designed to specifically address changes in symptoms of COPD with tiotropium using sensitive, validated instruments.

The safety profile of tiotropium in a primary care COPD population was consistent with published data. The most frequently reported drug-related adverse event that was higher in the tiotropium group was notably dry mouth.

In conclusion, the results of this trial support the efficacy and safety of tiotropium 18 μg via the HandiHaler^® ^in a representative primary care-managed COPD population. Responses did not appear to be affected by either disease severity or the broad treatment at baseline. Results from this primary care-based trial were consistent with findings in a secondary care setting, though further studies of longer duration are required to confirm our findings.

## Competing interests

Daryl Freeman has no shares in pharmaceutical companies. She has received speaker's honoraria for speaking at sponsored meetings from the following companies marketing respiratory products: Altana, Boehringer Ingelheim (BI), GlaxoSmithKline (GSK). She has received honoraria for advisory panels with: Altana, BI and GSK, and assistance with research projects from AstraZeneca, BI and GSK. Daryl Freeman receives funding for a clinical post from AstraZeneca, BI and GSK and has recently been funded to attend an international conference by Altana.

Angela Lee has no shares in pharmaceutical companies. She was previously a permanent employee of BI and was the trial statistician on the SPRUCE study. She has been working as an independent consultant statistician for the past two and a half years.

David Price has no shares in pharmaceutical companies. He has received speaker's honoraria for speaking at sponsored meetings from the following companies marketing respiratory products: 3 M, Altana, AstraZeneca, BI, GSK, IVAX, Merck, Sharp & Dome (MSD), Novartis, Pfizer, Schering-Plough. He has received honoraria for advisory panels with; 3 M, Altana, AstraZeneca, BI, GSK, IVAX, MSD, Novartis, Pfizer, Schering-Plough. He or his research team have received funding for research projects from: 3 M, Altana, AstraZeneca, BI, GSK, IVAX, MSD, Novartis, Pfizer, Schering-Plough, Viatris.

## Authors' contributions

All authors participated in the design of the study, interpretation of data and the drafting and approval of the final manuscript. AL performed the statistical analyses.

## Participating investigators

Daryl Freeman; Rupert Jones; Chris Woodforde; Teck L Lee; Lesley Starr; Deborah Beale; Janice Patrick; Kevin Gruffydd-Jones; Ian Parker; Nick Jones; John Tilley; A Gabriel; Ian Orpen; Tom Maxwell; Bryan Hopwood; Bhavesh Bodalia; Mark Reid; Alan Jones; Alan A Jones; Emyr Davies; Anne Weaver.
